# Old Drug, New Delivery Strategy: MMAE Repackaged

**DOI:** 10.3390/ijms24108543

**Published:** 2023-05-10

**Authors:** Hanane Lahnif, Tilmann Grus, Evangelia-Alexandra Salvanou, Elisavet Deligianni, Dimitris Stellas, Penelope Bouziotis, Frank Rösch

**Affiliations:** 1 Department of Chemistry—TRIGA Site, Johannes Gutenberg University Mainz, 55128 Mainz, Germanyfroesch@uni-mainz.de (F.R.); 2 Radiochemical Studies Laboratory, INRASTES, National Center for Scientific Research “Demokritos”, Ag. Paraskevi, 15341 Athens, Greece; 3 Institute of Chemical Biology, National Hellenic Research Foundation, 11635 Athens, Greece

**Keywords:** MMAE, PSMA, drug targeting, small molecule–drug conjugates, prostate cancer, therapeutic efficacy

## Abstract

Targeting therapy is a concept that has gained significant importance in recent years, especially in oncology. The severe dose-limiting side effects of chemotherapy necessitate the development of novel, efficient and tolerable therapy approaches. In this regard, the prostate specific membrane antigene (PSMA) has been well established as a molecular target for diagnosis of, as well as therapy for, prostate cancer. Although most PSMA-targeting ligands are radiopharmaceuticals used in imaging or radioligand therapy, this article evaluates a PSMA-targeting small molecule–drug conjugate, and, thus, addresses a hitherto little-explored field. PSMA binding affinity and cytotoxicity were determined in vitro using cell-based assays. Enzyme-specific cleavage of the active drug was quantified via an enzyme-based assay. Efficacy and tolerability in vivo were assessed using an LNCaP xenograft model. Histopathological characterization of the tumor in terms of apoptotic status and proliferation rate was carried out using caspase-3 and Ki67 staining. The binding affinity of the Monomethyl auristatin E (MMAE) conjugate was moderate, compared to the drug-free PSMA ligand. Cytotoxicity in vitro was in the nanomolar range. Both binding and cytotoxicity were found to be PSMA-specific. Additionally, complete MMAE release could be reached after incubation with cathepsin B. In vivo, the MMAE conjugate displayed good tolerability and dose-dependent inhibition of tumor growth. Immunohistochemical and histological studies revealed the antitumor effect of MMAE.VC.SA.617, resulting in the inhibition of proliferation and the enhancement of apoptosis. The developed MMAE conjugate showed good properties in vitro, as well as in vivo, and should, therefore, be considered a promising candidate for a translational approach.

## 1. Introduction

Chemotherapy is one of the most important pillars in the treatment of cancer diseases. However, the toxicity of this approach, resulting from the unspecific interaction of cytotoxic drugs with healthy tissue, presents one of the major drawbacks of this cancer treatment [1,2].

Targeted drug delivery is one of the strategies developed in recent decades to face this challenge. Antibody–Drug Conjugates (ADCs) represent a breakthrough [3,4]. An ADC consists of a cytotoxic drug and an antibody with high affinity to its oncological target. Both components are connected via a linker unit, which should be easily cleavable. Since ADCs are supposed to be stable constructs, the cytotoxic payload is only released after binding to the target. Nevertheless, this approach seems to have some limitations and disadvantages, such as a long circulation time and, therefore, high exposure for healthy tissues, reduced EPR effect, due to low penetration of the large antibodies into tumor tissue, and expensive and sophisticated synthesis of the drug conjugates [4,5,6,7]. Small molecule–drug conjugates (SMDCs) have been developed to tackle some of these challenges. Their low molecular weight enables good cell penetration in solid tumors. Moreover, as most SMDCs are smaller than 40 kDa, they are rapidly excreted from the blood through glomerular filtration, and, thus, display lower off-target cytotoxicity than ADCs. Other noteworthy facts concerning SMDCs are the simplified and manageable synthesis, compared to the sophisticated manufacturing process of monoclonal antibodies (mAbs), as well as the more straightforward transportation and storage processes, which are of immense value in regard to translational use of the corresponding drug conjugates [8,9].

Prostate cancer is the most common cancer in men and the second-leading cause of cancer death worldwide [10,11]. In the management of prostate cancer, the prostate-specific membrane antigen (PSMA) has been validated as a reliable tumor-associated biomarker and target for diagnosis, as well as for therapy of this disease [12,13]. PSMA is a membrane glycoprotein expressed on prostate tumor cells at significantly higher concentrations than on healthy tissue. Additionally, PSMA expression correlates with the grade of metastasis and progression of the tumor, allowing concise disease staging and therapy [14,15,16].

In the last decade, several PSMA-targeting pharmaceuticals have been developed. Small molecule, radio-labeled PSMA inhibitor-based radiopharmaceuticals, such as [^68^Ga]Ga-PSMA-11 and [^177^Lu]Lu-PSMA-617, have played significant roles in the tremendous progress nuclear medicine has experienced in the diagnosis and therapy of prostate cancer [17,18,19,20]. However, only a few PSMA-targeted drug conjugates have been investigated so far.

Targeting PSMA via mAb was the strategy adopted in the development of MLN2704, which was the first clinically tested PSMA-ADC. MLN2704 consists of the mAb J591 conjugated to the microtubule inhibitor maytansinoid 1 via a redox-sensitive disulfide linker. Although MLN2704 displayed satisfying results in a phase 1 clinical trial conducted with 23 patients, further application was discontinued after conducting a phase 2 large cohort study, due to severe adverse effects, such as neurotoxicity. It was assumed that the instability of the ADC and the resulting premature release of the cytotoxic drug were the major reasons for this negative outcome [21]. PSMA–MMAE is another PSMA-targeting ADC recently investigated in several clinical trials. The main component of PSMA–MMAE is the fully human mAb IgG1, which is conjugated to the antimitotic drug MMAE through a protease cleavable linker [22]. Although the results of its phase 2 clinical trial, reported in 2020, indicated lower neurotoxicity than MLN2704, instability of the drug conjugates, and subsequent deconjugation, were the major limitations of further clinical application, even though an enzyme cleavable linker was used [23,24]. A similar fate was faced by MEDI3726, a PSMA–ADC with high specific cytotoxicity in vitro but insufficient tolerability in patients. Further development was discontinued after the outcomes of the phase 1 study [25]. Generally, it can be concluded that the investigated PSMA–ADCs have failed, so far, in delivering the cytotoxic payload specifically to the tumor, due to their instability in blood circulation and the subsequent premature drug release. One possible approach to avoid these pitfalls is the development of small molecule–drug conjugates through replacement of the mAb in ADCs with small molecule inhibitors. Inspired by the successful implementation of this approach in PSMA radiopharmaceuticals, several efforts have been, and continue to be, made to apply the lessons learned from PSMA research.

Roy et al. [26] described a PSMA–SMDC consisting of the high affinity targeting moiety DUPA, a disulfide linker and an indenoisoquinoline topoisomerase I inhibitor. Additionally, a peptide linker was used to improve the hydrophilicity and the orientation of the SMDC within the PSMA binding pocket. The DUPA–drug conjugate displayed high binding affinity in vitro along with antitumor efficacy and good tolerability in xenograft models. Based on these promising results, another DUPA–SMDC was developed using paclitaxel as the cytotoxic drug in addition to a disulfide linker to ensure tumor specific release. DUPA–PTX also achieved encouraging results in cell assays and animal studies [27]. In 2019, a research group, from the company Endocyte, published preclinical results for EC1169, the first PSMA–SMDC to enter clinical trials. EC1169 is composed of the KuE–PSMA binding unit, the antimitotic drug tubulysin B hydrazide and a disulfide-based linker. It showed outstanding features in terms of good binding affinity in vitro, and high therapeutic efficacy and safety in vivo compared to docetaxel, which is the standard of care chemotherapeutic agent in the management of mCRPC. This favorable profile encouraged further testing in a phase 1 clinical trial [28].

Wang et al. presented the results obtained from preclinical studies of a novel PSMA targeting SMDC. PSMA-1–VcMMAE was developed, based on PSMA–ADC through replacement of the mAb by the small molecule PSMA-1. Both PSMA drug conjugates use MMAE as the cytotoxic drug and the cathepsin cleavable linker valine–citrulline. However, PSMA–ADC displayed higher cytotoxic potency in PSMA-positive cells, probably due to the higher affinity of the mAb to PSMA. Nevertheless, animal studies proved the superiority of PSMA-1–VcMMAE over PSMA–ADC in terms of a larger therapeutic index [29]. A similar approach was applied by Boinapally et al. [30], who recently reported the development of SBPD-1 which consists of MMAE, valine–citrulline linker and a small-molecule PSMA-binding unit. SBPD-1 demonstrated high binding affinity in the low-nanomolar range along with PSMA-dependent cytotoxicity and antitumor effect in xenograft models. The tolerability and, thus, the translational potential of this SMDC were the main highlighted benefits.

In summary, the herein described SMDCs (Table 1) have to prove their translational potential by undergoing clinical testing. However, it will be exciting to see whether they succeed in circumventing the drawbacks of ADCs to, thus, avoid a similar fate.

**Figure 1:**
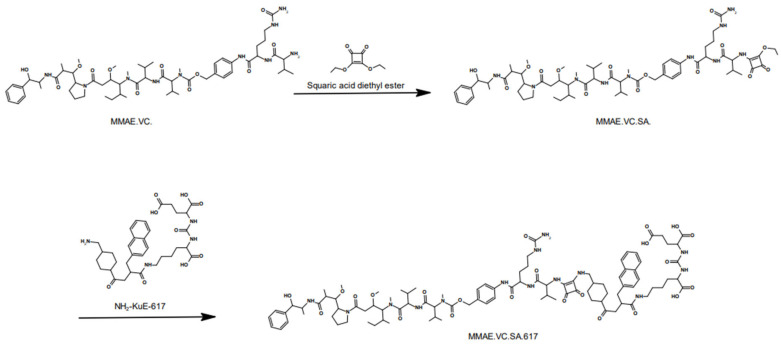
Synthesis of MMAE.VC.SA.617. In the first step, the primary amine of the terminal valine of MMAE.VC was conjugated to squaric acid diethyl ester through an asymmetric amidation in an aqueous phosphate buffer at pH 7. To improve the solubility of MMAE.VC, DMSO was added. The reaction resulted in a quantitative conversion of MMAE.VC to MMAE.VC.SA monitored by LC-MS. In the second step, MMAC.VC.SA, as well as NH2-KuE-617, were dissolved in ethanol and, in the presence of triethylamine, the second asymmetric amidation took place. MMAC.VC.SA.617 was isolated in a 43% yield by semi-preparative HPLC purification.

Developing a small molecule drug conjugate requires a thorough design and the right choice of building components. The SMDC described in this study consists of three main modules. The targeting unit KuE-617 refers to the combination of the PSMA-inhibitor KuE (lysine-urea-glutamate) and the naphthyl-cyclohexyl linker of PSMA-617. The chemical structure of these compounds is shown in Figure 1. This moiety is crucial in the design of targeted drug delivery systems since it is responsible for delivering the cytotoxic payload to the desired “address”. Obviously, this cytotoxic payload needs to be a highly potent drug, mostly with a narrow therapeutic window and, therefore, not applicable as a single drug. Herein, the potent antimitotic drug of choice was Monomethyl auristatin E (MMAE), which is one of several ADCs, such as the FDA-approved brentuximab vedotin (ADCETRIS™). Besides the essential components, the linker plays a decisive role in the efficacy, stability and resulting tolerability of the whole SMDC. In the developed SMDC, a valine–citrulline linker was used to conjugate MMAE to KuE-617. This dipeptide belongs to the class of enzyme-cleavable linkers. It is cleaved by cathepsin B, which is a lysosomal protease overexpressed in various forms of cancer, including prostate cancer [31,32,33,34].

## 2. Results

### 2.1. Synthesis

The synthesis of MMAE.VC.SA.617 was carried out in a fast and straightforward two-step synthesis. The commercially available compounds MMAE.VC and NH_2_-KuE-617 were used as starting components and were coupled using squaric acid diester as the linking unit. The synthesis route is shown in Figure 1.

The use of the squaric acid diester as a coupling reagent is particularly suitable because of its ability to conjugate two amines quickly, selectively and under mild conditions. This asymmetric amidation can be performed in both aqueous and organic media, which makes it very versatile. It is highly selective towards amines, making the use of protecting groups on other nucleophilic groups unnecessary. This coupling method is receiving more and more attention, ranging from the conjugation of bioconjugates to nanoparticles to be used in radiopharmaceuticals [35,36,37].

### 2.2. Binding Affinity

**Figure 2:**
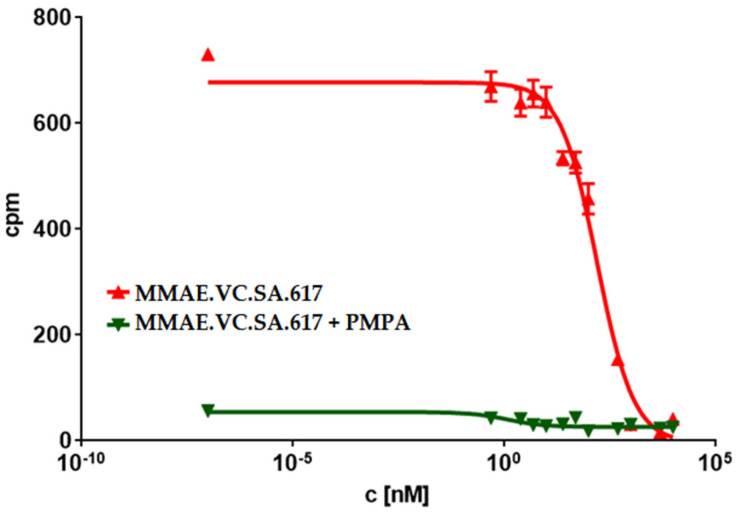
Concentration-inhibition curve of MMAE.VC.SA.617 compared to MMAE.VC.SA.617 + PMPA (n = 3). Note: cpm: counts per minute.

One of the most important characteristics of drug conjugates is the specific and strong interaction with the addressed oncological target. In order to evaluate the binding potency of the KuE-617/MMAE-conjugate for PSMA, we performed a competitive radioligand binding assay using PSMA-positive LNCaP cells. Furthermore, we determined the affinity of the drug-free KuE-617 in the same assay to get a better understanding of the effect of conjugation regarding the interaction within the PSMA binding pocket. The measured binding affinity of KuE-617, expressed as the IC_50_ value, was in the same range as the clinically used PSMA radiopharmaceuticals [^68^Ga]Ga-PSMA-11 and [^617^Lu]Lu-PSMA-617. However, the insertion of MMAE decreased the binding potency (Table 2). It should be noted that the binding of MMAE.VC.SA.617 was demonstrated to be PSMA-specific, since no binding occurred after co-incubation with the potent PSMA inhibitor 2-(Phosphonomethyl) pentanedioic acid PMPA, which acted as a blocking agent at the PSMA receptor (Figure 2).

### 2.3. In Vitro Cytotoxicity

#### 2.3.1. CellTiter-Blue^®^ Viability Assay

The cytotoxicity of MMAE.VC.SA.617 was evaluated in vitro using Celltiter-Blue^®^. The conjugation of MMAE to KuE-617, resulting in the dimeric compound, seems to affect the in vitro cytotoxicity, since the IC_50_ value of the MMAE conjugate was approximately 100 times higher than that of the non-conjugated MMAE. Blockade of PSMA receptors by excess addition of PMPA led to a decrease in the cytotoxic effect of MMAE.VC.SA.617. Furthermore, co-incubation of the LNCaP cells with the cathepsin inhibitor E-64 led to a similar decrease in cytotoxicity. This is probably due to the inhibition of cathepsin B, which is responsible for cleavage of the valine–citrulline linker and, thus, the subsequent release of MMAE. Table 3 shows the results of the Celltiter-Blue^®^ cytotoxic assay.

#### 2.3.2. Immunofluorescence Studies

**Figure 3:**
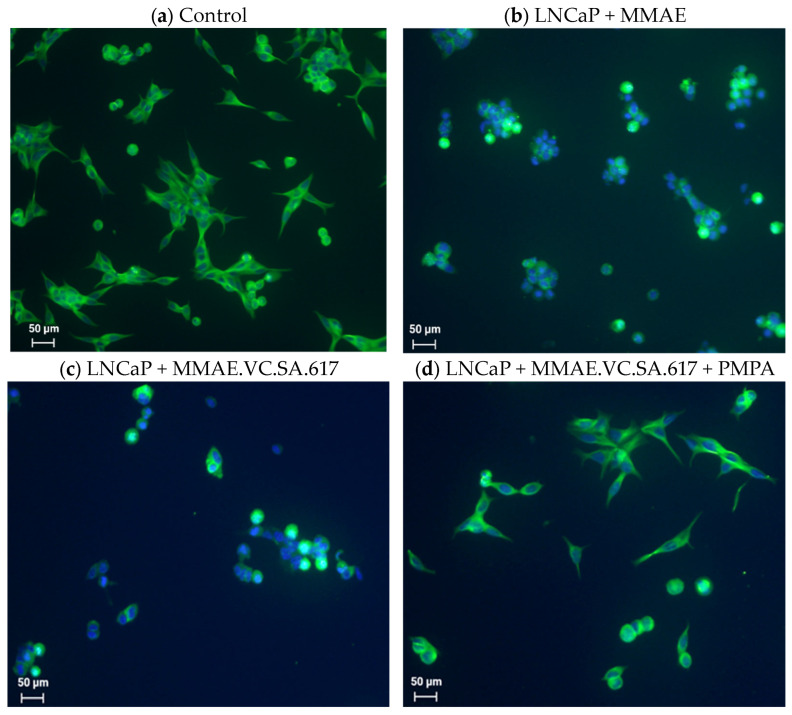
Immunofluorescent staining of α-tubulin (green) with Alexa fluor 488 α-tubulin antibody. Cell nuclei were counterstained with DAPI (blue). Images were taken at 20× magnification. LNCaP cells were used as control (**a**), incubated with either 1 nM MMAE (**b**), or 100 nM MMAE.VC.SA.617 (**c**). PSMA-specific effect was determined by co-incubation of 100 µM PMPA (**d**).

To further evaluate the cytotoxic effect of MMAE, compared to MMAE.VC.SA.617, LNCaP cells were treated for 24 h with either MMAE (Figure 3b), MMAE.VC.SA.617 (Figure 3c) or MMAE.VC.SA.617 with the addition of PMPA (Figure 3d) prior to co-staining with DAPI (4′,6-diamidino-2-phenylindole) and α-tubulin antibody. Cells incubated with both MMAE and MMAE.VC.SA.617 showed distinctive tubulin disruption resulting in substantial damage of the microtubule cytoskeleton. However, co-incubation with PMPA reduced the cytotoxic effect of MMAE.VC.SA.617 which, again, demonstrated the PSMA selectivity of this SMDC.

### 2.4. Cathepsin B Cleavage Assay

**Figure 4:**
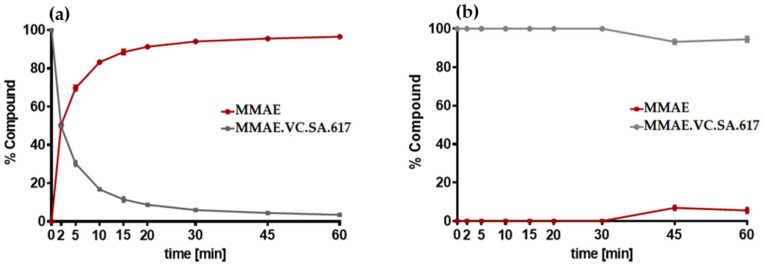
(**a**) Quantification of MMAE release after incubation with cathepsin B at 37 °C; (**b**) control experiment: stability of MMAE.VC.SA.617 in PBS without incubation with cathepsin B.

The cathepsin-specific cleavage of MMAE.VC.SA.617 was evaluated by incubating LNCaP cells at 37 °C with cathepsin B (Figure 4a). A control experiment was conducted by determining the stability of MMAE.VC.SA.617 in PBS in the absence of cathepsin (Figure 4b). Aliquots were withdrawn at different time points and analyzed via liquid chromatography mass spectrometry LC/MS. The concentration of free MMAE increased continuously after incubation with cathepsin B. Complete release from the drug conjugate was already reached after approximately 20 min. In contrast, in the absence of cathepsin B, MMAE.VC.SA.617 remained almost stable.

### 2.5. Animal Studies

#### 2.5.1. Toxicity and Therapeutic Efficacy Studies

**Figure 5:**
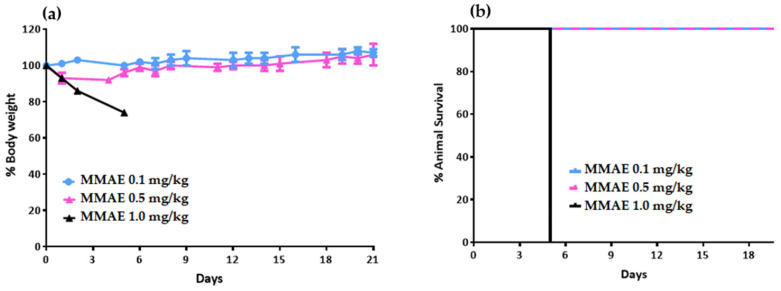
Body-weight change (**a**) and survival curve (**b**) of NOD/SCID mice after one intravenous (i.v.) injection of 1 mg/kg MMAE (n = 3 mice per group).

In order to specify the pharmacological properties of MMAE.VC.SA.617 we inoculated LNCaP cells into NOD/SCID mice to generate a xenograft model. Prior to in vivo studies in tumor-bearing mice, we conducted a toxicological study in healthy NOD/SCID mice to determine the maximal tolerable dose of MMAE. Mice injected with 1 mg/kg MMAE had to be euthanized 5 days post-injection, in accordance with humane endpoint criteria, due to body weight loss of over 20% (Figure 5).

**Figure 6:**
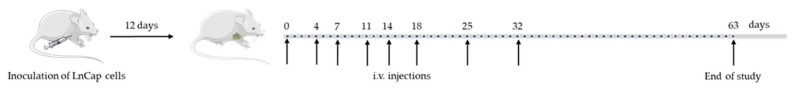
Timeline of therapeutic protocol and assessment of therapeutic outcome. LNCaP cells were inoculated into NOD/SCID mice. At different time points mice were injected with 0.1 mg/kg, 0.5 mg/kg or 1.0 mg/kg MMAE.VC.SA.617 in the verum group or. 0.1 mg/kg and 0.5 mg/kg MMAE in the reference group. Mice in the control group received 0.9% NaCl.

**Figure 7:**
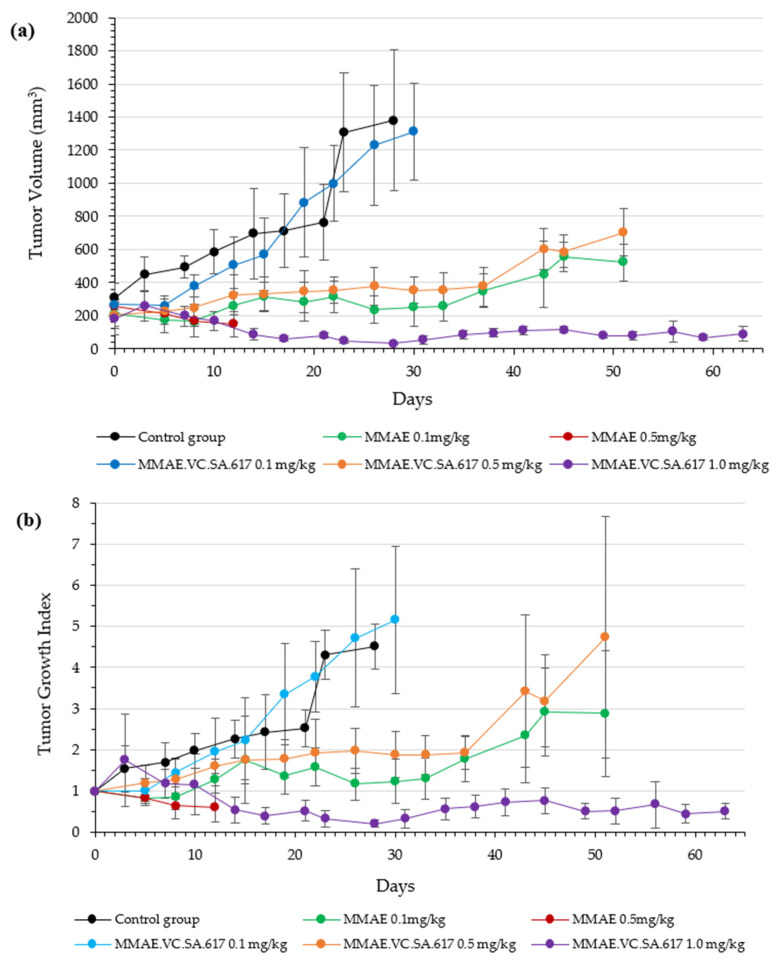

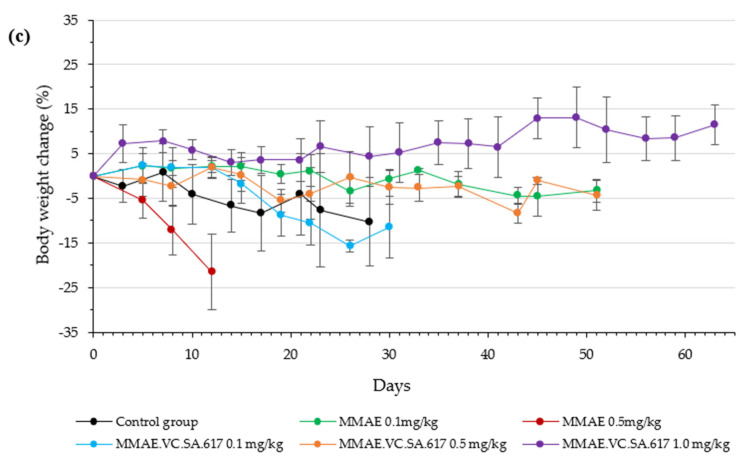
Therapeutic efficacy study of MMAE and MMAE.VC.SA.617 in LNCaP prostate cancer xenografts. Mice were intravenously injected through the tail vein. Treatments were performed on day 0, 4, 7, 11, 14, 18, 25 and 32 (eight doses). Each group had 4 NOD/SCID mice. The plots of each group have different endpoints corresponding to animal death. (**a**): Tumor Volume; (**b**): Tumor Growth Index (TGI); (**c**): Percentage body weight change of mice.

**Figure 8:**
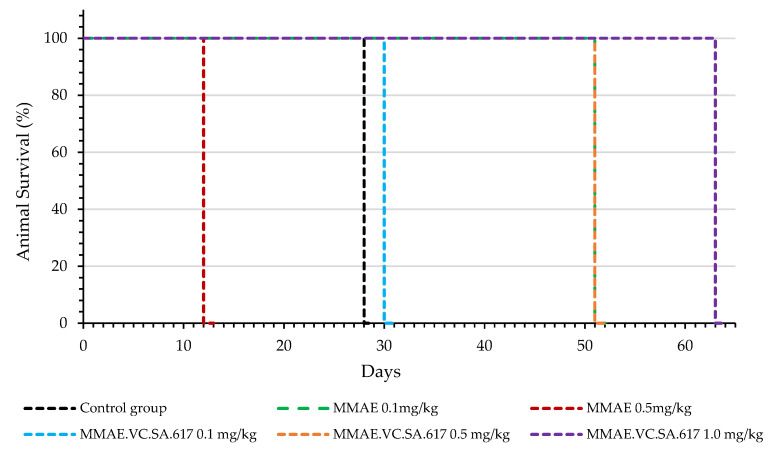
Kaplan–Meier survival curves of NOD/SCID mice. The mice of the 1.0 mg/kg MMAE.VC.SA.617 therapy group survived until the end of the experiment (Day 63 of treatment). On the contrary, the mice in the 0.5 mg/kg MMAE group had to be euthanized on day 12, because the repeated intravenous administrations of MMAE resulted in drastic decrease in body weight.

**Figure 9:**
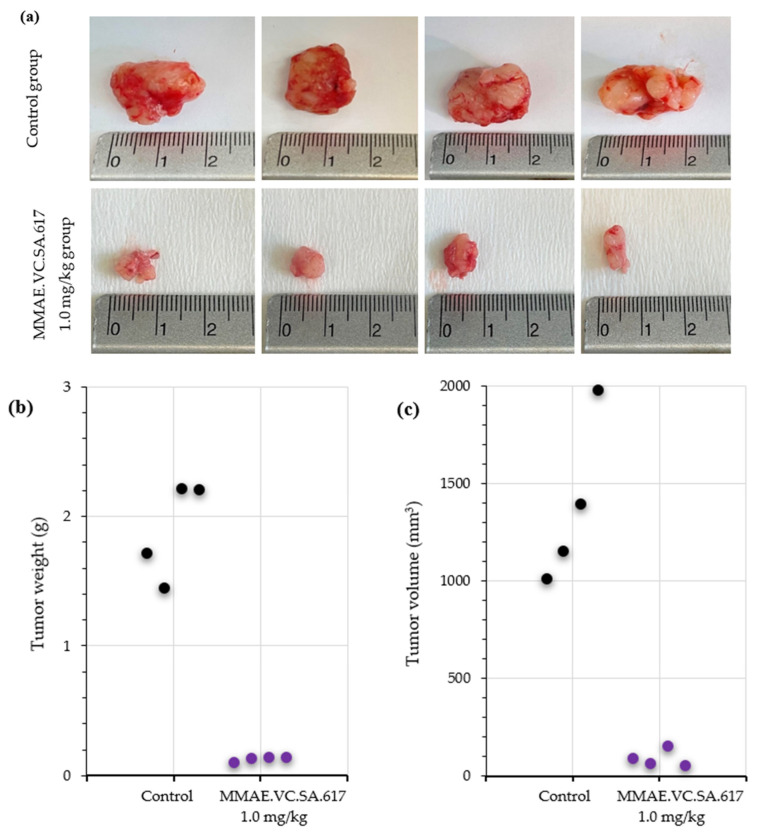
(**a**) Representative images of excised tumors: Upper panel—Control group of mice injected with 0.9% NaCl; Lower panel—Therapy group of mice injected with 1.0 mg/kg MMAE.VC.SA.617. Reduction in weight (**b**) and volume (**c**) of the tumors of the control group versus the 1.0 mg/kg MMAE.VC.SA.617 treated group.

Based on these findings, in vivo therapeutic efficacy studies were conducted with 3 different drug concentrations of MMAE.VC.SA.617, namely 0.1 mg/kg (corresponding to 0.05 mg MMAE), 0.5 mg/kg (corresponding to 0.25 mg MMAE) and 1.0 mg/kg (corresponding to 0.49 mg MMAE). The corresponding concentrations of administered MMAE were estimated by taking into account the molecular weight of MMAE.VC.SA.617 and MMAE (1451.77 g/mol and 718 g/mol, respectively). In the reference group, mice were injected with either 0.1 mg/kg or 0.5 mg/kg MMAE. All mice received 8 injections of either MMAE, MMAE.VC.SA.617 or 0.9% NaCl, according to Figure 6:

The results of the therapeutic efficacy study are presented both as Tumor volume and Tumor Growth Index (TGI) (Figure 7). The group of mice injected with plain MMAE 0.5 mg/kg were euthanized on day 12 of experimentation due to weight loss (≥20% weight loss, Figure 7 and Figure 8, red line). Although one mouse in this group had no evident palpable tumor after the 4th injection, the treatment led to augmented weight loss indicating the accumulative toxicity of therapy with this MMAE concentration. On the contrary, all animals survived in the 0.1 mg/kg MMAE group up to day 51 (Figure 7 and Figure 8, green line), where a moderate increase in tumor volume was observed. The mice of the 0.1 mg/kg MMAE.VC.SA.617 group survived until Day 30, which can be attributed to the low amount of MMAE which the mice in this group received (Figure 7 and Figure 8, blue line). In this group, tumor volume increased continuously over time, while in the group with the 0.5 mg/kg concentration of MMAE.VC.SA.617, treatment seemed to inhibit tumor growth resulting in a constant tumor volume up to day 37 (Figure 7, Panels A and B, orange line). In the 1.0 mg/kg MMAE.VC.SA.617 therapy group, the treated mice seemed to tolerate the treatment well, since body weight remained almost constant and all mice survived until the end of the experiment (Day 63 of treatment, Figure 7, purple line). In this group, tumor growth showed a continuous regression up to day 17, after which disease stabilization was evident. In a direct comparison of the MMAE.VC.SA.617 1.0 mg/kg group with the MMAE 0.5 mg/kg group, mice in both groups showed remarkably similar behavior in tumor volume development, which may be attributed to the fact that both groups received a similar amount of MMAE per injection (0.49 mg vs. 0.50 mg MMAE, respectively). However, the mice in the 0.5 mg/kg MMAE group had to be euthanized on day 12, because the repeated intravenous administrations of MMAE were toxic, while repeated administrations of 1.0 mg/kg MMAE.VC.SA.617 did not seem to affect the overall well-being of the mice (stable body weight, no signs of discomfort) and led to tumor regression. Substantial tumor growth inhibition for the 1.0 mg/kg MMAE.VC.SA.617 group, in comparison to the control group, is clearly visualized in Figure 9.

#### 2.5.2. Tumor Histology

**Figure 10:**
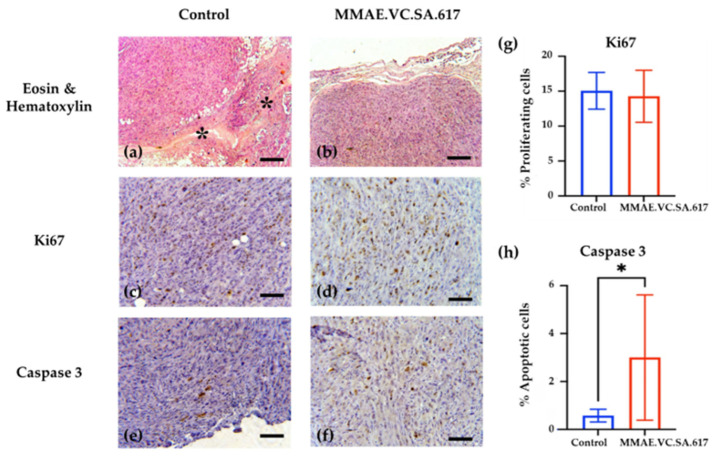
Representative images of the control and MMAE.VC.SA.617-treated tumors (1 mg/kg body weight). Panel (**a**,**b**) indicate the histology of the tumors stained with Hematoxylin and Eosin. The stars in (**a**) indicate the central necrotic area of the tumors. In (**c**,**d**) the Ki67 staining was visualized with DAB (3,3′-Diaminobenzidine) indicating similar growth rates in the periphery of the tumors. In (**e**,**f**) the caspase 3 staining, also visualized with DAB, indicate a higher apoptotic rate of the treated tumors. In (**g**,**h**) the graphs show the actual % percentages of the proliferating and apoptotic cells respectively. Scale bar 300 μm. * *p* < 0.05.

The overall histology of the tumors revealed similarities between the treated and the control tumors. We observed a central necrotic area, which was anticipated for the bigger tumors due to the lack of sufficient vasculature. Similarly, but to a lesser extent, these necrotic features were also present in the smaller treated tumors. We decided to analyze the apoptotic rate of cancer cells and their proliferation status, using cleaved caspase-3 and Ki67 staining, respectively. For that purpose, we evaluated the staining in the periphery of the tumors and not on the central necrotic part (Figure 10, Panels a–f). Our results showed that the proliferating cells in the periphery of the tumors had similar proliferating rates. To be more specific, 15.06% of the control and 14.27% of the treated tumors stained positive for Ki67 (Figure 10g). On the contrary, we observed an increase of the apoptotic cells in the treated tumors. The caspase 3 staining revealed a significant increase of the apoptotic cells (*p* = 0.023) located in the periphery of the treated tumors. The mean value of apoptotic cells was 3.002%. The control tumors showed very little apoptotic percentage on the periphery, with a mean value of apoptotic percentage of 0.579% (Figure 10h).

## 3. Discussion

The development of strategies for selective drug delivery is one of the most important research fields in the fight against cancer. Herein, we described a small-molecule drug conjugate consisting of the potent antimitotic drug MMAE and the high affinity PSMA inhibitor derivative KuE-617. Both entities were linked via a valine–citrulline linker. The design, synthesis and subsequent in vitro and in vivo evaluations were performed in several steps.

The compound MMAE.VC.SA.617 was synthesized in a fast and straightforward two-step synthesis. The two units of the compound, the drug-linker conjugate MMAE.VC and the PSMA-binding unit KuE-617, were conjugated via asymmetric amidation using a squaric acid linking unit. In the first step, squaric acid had to be added every second day, as it was observed, via LC–MS, to be consumed during the reaction. After eight days, MMAE had been quantitatively converted. In this case, the amidation was carried out in an aqueous buffer solution, as the progress of the reaction was controlled by the pH value. However, since the MMAE conjugate did not dissolve completely in water, DMSO was added. The second stage of the reaction was carried out in ethanol. In the organic medium, triethylamine was added as a base to enable the second asymmetric amidation of the squaric acid linker unit. The final compound MMAE.VC.SA.617 was obtained after semi-preparative HPLC purification.

The PSMA-binding affinities of MMAE.VC.SA.617, as well as of the drug-free conjugate KuE-617, were determined in a cell-based radioligand competitive assay. The IC_50_ value of KuE-617 was in the low nanomolar range, similar to the chelator-based PSMA radioligands PSMA-617 and PSMA-11 (21.5 ± 1.9 nM, 15.1 ± 3.8 nM and 17.4 ± 1.6 nM respectively). However, the insertion of MMAE led to a significant decrease in PSMA-binding affinity resulting in a nine-fold higher IC_50_ value than that of KuE-617 (188.6 ± 24.7 nM vs. 21.5 ± 1.9 nM), indicating that the conjugation of KuE-617 to MMAE had a negative impact on the binding affinity of the PSMA inhibitor, probably due to changes in the conformation of the drug conjugate and its orientation within the PSMA binding pocket.

In the field of drug targeting, selecting a potent drug is as important as designing a high affinity targeting unit. The cytotoxic drug used herein was the tubulin inhibitor MMAE, which is already used in several ADCs [38,39,40]. To characterize the pharmacological properties of the MMAE conjugate we determined its cytotoxicity using CellTiter-Blue^®^. This assay is based on the ability of viable cells to transform resazurin into the fluorescence-emitter resorufin. Thus, the fluorescence signal correlates with cell viability. The IC_50_ value of the single drug MMAE was, as expected, in the picomolar range, whereas the cytotoxicity of MMAE.VC.SA.617 was about 100-fold lower (0.23 ± 0.06 nM vs. 33.0 ± 4.9 nM). This could be related to a potential decrease in lipophilicity of MMAE.VC.SA.617 due to the added carboxylic groups of the KuE unit and the ureido group of citrulline, resulting in a reduction in passive diffusion through the cell membrane.

Another possible reason could be incomplete release of MMAE within tumor cells as a result of a low cathepsin B level in LNCaP cells or even an impaired internalization ratio. Nevertheless, the PSMA-specific uptake of MMAE.VC.SA.617 could be demonstrated by blocking PSMA receptors using PMPA which led to a three-times lower cytotoxicity (92.8 ± 8.3 nM). Additionally, the inhibition of cathepsin B via co-incubation with E-64 and the resulting decrease in cytotoxicity proved the essential role that this enzyme plays in the cleavage of the valine–citrulline linker and the subsequent release of MMAE. This enzyme-dependent cleavage of the dipeptide linker is a crucial feature in targeted therapeutics, since the active drug should be released only after uptake in tumor cells.

In a further step, we tried to further characterize the cytotoxicity of MMAE.VC.SA.617 using immuno-fluorescence imaging. It is known that the antimitotic drug MMAE acts by inhibiting the α-tubulin polymerization. This was proved with the conducted immunofluorescence studies, which showed a distinctive disturbance in tubulin formation of LNCaP cells incubated with either MMAE or MMAE.VC.SA.617 (Figure 3b,c). The cytotoxic effect of MMAE.VC.SA.617 could be reduced by co-incubation with PMPA, which led to blocking of PSMA receptors (Figure 3d). These results were in accordance with the findings described above.

The targeted delivery of cytotoxic drugs to tumor cells requires not only binding to tumor-associated structures but also a specific release of the conjugated drug in tumor tissue. The SMDC described herein included a valine–citrulline linker, which is one of the commonly used linkers in ADCs [31,32]. Valine–citrulline is cleaved by enzymes of the cathepsin family, especially cathepsin B, which is highly expressed in tumor cells [33,34]. In order to verify the cathepsin-specific cleavage of MMAE.VC.SA.617, the ratio of MMAE over time in the presence or absence of cathepsin B was quantified (Figure 4a,b). As expected, MMAE was completely released after about 20 min of incubation with cathepsin B, whereas MMAE.VC.SA.617 remained almost stable in PBS.

Based on the positive results obtained from the in vitro assays, animal studies were performed in order to characterize the in vivo profile of MMAE.VC.SA.617 in terms of antitumor effect and tolerability. The single drug MMAE was used as the reference. According to the results from the initial toxicity study, three different concentrations of MMAE.VC.SA.617 were selected: 0.1 mg/kg corresponding to 0.05 mg MMAE, 0.5 mg/kg corresponding to 0.25 mg MMAE and 1 mg/kg MMAE.VC.SA.617, corresponding to 0.49 mg/kg MMAE. This MMAE concentration range was found to be well-tolerated by the mice during the initial toxicity study. Twelve days after inoculation of the mice with LNCaP tumor cells, treatment was initiated, according to Figure 6 (shown above). All five treatment groups received therapy on days 0 (first day of treatment), 4, 7, 11, 14, 18, 25 and 32. The control group of mice received 0.9% NaCl on the same days.

Animals treated with 0.1 mg/kg MMAE survived until day 51 of treatment and showed a constant tumor volume; however, the overall condition of the mice had deteriorated, and, thus, they had to be euthanized in accordance with bioethics principles (Figure 7 and Figure 8). On the other hand, the mice in the 0.5 mg/kg MMAE group had to be euthanized by day 12 of the treatment due to approximately 20% weight loss, indicating severe toxicity of the single drug MMAE. The mice treated with 0.1 mg/kg MMAE.VC.SA.617 were euthanized before the end of the experiment due to body weight loss and large tumor volume. These results could be attributed to insufficient drug delivery into the tumor tissue. As concluded from in vitro studies the cytotoxicity of the MMAE conjugate was ten-fold lower in magnitude than the single drug MMAE, as shown above. This loss in cytotoxicity could not be compensated by active PSMA-targeting since MMAE.VC.SA.617 displayed moderate PSMA-binding affinity. Despite these results for the 0.1 mg/kg MMAE.VC.SA.617 group, tolerability to MMAE.VC.SA.617 of the two other concentrations (0.5 mg/kg and 1.0 mg/kg) was demonstrated (100% animal survival) even after eight intravenous injections. Moreover, tumor growth was effectively inhibited. The 0.5 mg/kg MMAE.VC.SA.617 treatment dose was tolerated well in terms of tumor volume and body weight; however, as in the case of the 0.1 mg/kg MMAE mice, the mice had to be euthanized due to poor body score. The mice which were administered with 1 mg/kg MMAE.VC.SA.617 showed impressive tumor regression after the second therapeutic administration (day 3 of therapy), which remained practically stable until the end of the experiment (day 63).

The analysis of the histopathologic features of the tumors revealed increased necrotic areas, mostly located in the central area of the tumors. The control tumors showed extensive necrotic areas, which were attributed to the lack of vascularization. On the contrary, both the treated and the control tumors showed normal proliferating rates on the periphery. However, treatment with MMAE.VC.SA.617 resulted in a six-fold higher apoptotic rate compared to the control group (3.002% vs. 0.579%), indicating an impact on either the apoptotic rate of the cancer cells or their proliferating status, or both. The smaller tumors collected from the treated animals indicated a robust inhibition of their growth, which could be explained either via direct killing of the cancer cells or via inhibition of their proliferation. These robust effects of the treatment might have been ameliorated at the end of the experiment, which was almost a month after the last injection of MMAE.VC.SA.617, and, thus, the proliferation and apoptosis that we observed reflected their current status and not the initial anti-tumoral function of the treatment.

Finally, MMAE.VC.SA.617 showed high binding affinity and cytotoxicity in vitro; in the nanomolar range for both. Although the single agent MMAE exhibited almost 100-fold higher cytotoxicity than the single drug in vitro, the SMDC clearly showed its superiority in vivo. Treatment with 0.5 mg/kg MMAE resulted in significant side effects, and animals had to be euthanized several days after intravenous injection. In contrast, mice treated with the same amount of MMAE, this time as SMDC, showed tumor regression after the second injection, even over the entire experimental period of more than 60 days, with a survival rate of 100%.

In general, targeting PSMA by small molecules has been shown to be superior to the use of antibodies. All PSMA ADCs investigated to date, such as MLN2704, PSMA-ADC or MEDI3726, failed in clinical trials due to serious adverse effects resulting from premature drug release [21,22,23,24,25,29]. Inspired by the successful implementation of small molecule PSMA radiopharmaceuticals, several PSMA SMDCs were developed. EC1169 is probably the best-known member of this group, as it was the first to be investigated in clinical trials. EC1169, which is composed of the KuE–PSMA binding unit, the antimitotic drug tubulysin B hydrazide and a disulfide-based linker, showed outstanding features in terms of good binding affinity in vitro, high therapeutic efficacy and safety in vivo [28,41]. However, the results of phase 1 studies results did not reveal sufficient efficacy. PSMA–SMDC and DUPA–PTX are two other PSMA drug conjugates containing a disulfide linker. Although both compounds showed good results in vitro and in vivo, they failed to confer any advantage over therapy with the unconjugated drug [26,27]. These results might indicate that the use of the disulfide linker does not lead to the desired specific drug release and, thus, does not achieve the expected efficacy, especially with regard to the positive results of the two valine–citrulline based SMDCs, PSMA-1–VcMMAE and SBPD-1. Both conjugates showed promising properties in terms of in vitro binding affinity and antitumor effect in xenograft models [29,30].

Thus, in accordance with our findings, the use of MMAE as the cytotoxic payload and valine-citrulline as the linker was shown to be beneficial in terms of efficacy and tolerability. However, direct comparability between MMAE.VC.SA.617 and the reported compounds cannot be made, since the in vitro and in vivo studies conducted with both PSMA-1VcMMAE and SBPD-1 were based on the use of PC3 PIP cells. This cell line was transduced to overexpress PSMA to a significantly higher extent than the patient-derived LNCaP cells used for testing of MMAE.VC.SA.617 [42].

## 4. Materials and Methods

### 4.1. General

Chemicals were purchased from Sigma-Aldrich, Merck, Darmstadt, Germany, VWR, AcrosOrganics and TCI, Darmastadt, Germany. MMAE.VC was purchased from Hycultec GmbH, Beutelsbach, Germany and PSMA-617-NH_2_ from Huayi Isotopes Co. Haiyu town Jiangsu, China. Deuterated solvents for NMR spectra were acquired from Deutero GmbH, Kastellaun, Germany. Silica gel 60 F254 coated aluminum plates from Merck, Darmstadt, Germany were used for thin layer chromatography. NMR measurements were performed on an Avance III 600 spectrometer (600 MHz, 5 mm TCI CryoProbe sample head with z-Gradient and ATM and SampleXPress Lite 16 sample changer) from Bruker, Billerica, MA, USA. The LC/MS measurements were performed on an Agilent Technologies 1220 Infinity LC system coupled to an Agilent Technologies 6130B Single Quadrupole LC/MS system, Agilent Technologies GmbH, Waldbronn, Germany. Semi-preparative HPLC purification was performed on a 7000 series Hitachi LaChrom, Krefeld, Germany, using a semi-preparative LiChrospher 100 RP18 EC (250 × 10 mm) 5 µm column.

### 4.2. Organic Synthesis

#### 4.2.1. MMAE.VC.SA

MMAE.VC (20 mg, 0.018 mmol) was dissolved in 0.5 M phosphate buffer pH 7 (500 µL) and DMSO (500 µL). 3,4-Diethoxycyclobut-3-ene-1,2-dione (5 mg, 4 µL, 0.027 mmol) was added and stirred for 8 days. Every second day 0.5 eq of 3,4-Diethoxycyclobut-3-ene-1,2-dione was added. The solvent was removed via lyophilization and the product was used in the next step without purification.

MS (ESI+): 236.0 ([M + H]^+^/2), calculated for C_64_H_98_N_10_O_15_: 1246.72 [M]^+^.

#### 4.2.2. MMAE.VC.SA.617

MMAE.VC.SA (20 mg, 0.016 mmol) and PSMA-617-NH_2_ (10 mg, 0.016 mmol) were dissolved in ethanol (3 mL). Triethylamine (50 µL) was added and the reaction mixture was stirred for 6 days. The solvent was removed under reduced pressure. MMAE.VC.SA.617 was obtained as a white powder (14.2 mg, 43%) after HPLC purification (LiChrospher 100 RP18 EC (250 × 10 mm) 5 μL, flow rate: 5 mL/min, H_2_O/MeCN + 0.1% TFA, 45% to 55% MeCN in 20 min, tR = 9.0 min).

MS (ESI+): 929.0 ([M + H]^+^/2), calculated for C_95_H1_37_N_15_O_23_: 1857.22 [M]^+^.

1H NMR (600 MHz, EtOD-*d*_6_) δ [ppm] = 7.77 (dd, J = 19.3, 9.2 Hz, 2H), 7.69 (d, J = 31.8 Hz, 3H), 7.41 (t, J = 7.9 Hz, 5H), 7.30 (dt, J = 15.4, 7.4 Hz, 3H), 7.24–7.15 (m, 1H), 5.18 (dt, J = 28.3, 15.2 Hz, 1H), 5.07 (d, J = 11.7 Hz, 1H), 4.93 (d, J = 31.6 Hz, 1H), 4.32–4.04 (m, 4H), 3.93–3.87 (m, 1H), 3.84 (s, 1H), 3.70 (s, 3H), 3.45 (d, J = 14.1 Hz, 2H), 3.41–3.26 (m, 9H), 3.23 (s, 1H), 3.11 (d, J = 15.3 Hz, 3H), 2.96 (td, J = 18.7, 7.0 Hz, 3H), 2.52 (s, 1H), 2.34–2.15 (m, 3H), 2.06 (s, 1H), 2.01–1.77 (m, 2H), 1.75–1.27 (m, 12H), 1.27–1.16 (m, 6H), 1.08–0.69 (m, 28H).

### 4.3. In Vitro Binding Affinity

LNCaP prostate cancer cells (purchased from Sigma-Aldrich, Darmstadt, Germany) were cultured in RPMI 1640 (Thermo Fisher Scientific, Dreieich, Germany) supplemented with 10% fetal bovine serum (Thermo Fisher Scientific), 100 μg/mL streptomycin, and 100 units/mL penicillin at 37 °C in 5% CO_2_.

LNCaP cells were incubated for 45 min with different concentrations of the MMAE-conjugates in the presence of 0.75 nM [^68^Ga]Ga-PSMA-10. Free radioactivity was removed by several washing steps with ice-cold PBS. Probes were measured in a γ-counter (2480 WIZARD2 Automatic Gamma Counter, PerkinElmer, Rodgau, Germany). Obtained data were analyzed in GraphPad Prism 9, GraphPad Software Boston, MA, USA, using nonlinear regression.

### 4.4. CellTiter-Blue^®^ Viability Assay

A total of 10^4^ cells per well were seeded in a 96-well plate for 24 h prior to incubation with increasing concentrations of either MMAE (0.1 nM to 0.5 µM) or MMAE.VC.SA.617 (2.5 nM to 10 µM). Subsequently, 20 µL of CellTiter-Blue^®^ Reagent were added in each well and incubated for 2 h at 37 °C. For blocking studies, 2.5 nmol of PMPA was added to each well prior to incubation with SMDC. Fluorescence (560^Ex^/590^Em^) was recorded using a Tecan Spark multimode reader.

### 4.5. Immunofluorescence Studies

A total of 2000 cells/well were seeded in a Nunc^®^ Lab-Tek^®^ II—CC2™ Chamber Slide™ (Sigma Aldrich) and incubated with the test compounds at 37 °C for 24 h. After fixation with 4% PFA, cells were permeabilized with 0.5% Triton X-100 for 15 min at room temperature. Cells were then washed several times with PBS and blocked with 3% BSA in PBS for 1 h at room temperature. α-tubulin staining was performed by incubating the cells with alpha-Tubulin Antibody, Alexa Fluor^®^ 488 conjugate (B-5-1-2) (Thermo Fisher Scientific) at a final concentration of 2 µg/mL for 3 h at room temperature. Counterstaining with DAPI was carried out with ProLong™ Gold Antifade Mountant with DAPI (Thermo Fisher Scientific), according to the manufacturer’s protocol. Cells were visualized using a fluorescence microscope (Keyence BZ-8000, KEYENCE DEUTSCHLAND GmbH, Neu-Isenburg, Germany) at 20×.

### 4.6. Cathepsin B Cleavage Assay

Cathepsin B from human liver (Sigma Aldrich) was activated by incubation at room temperature with 30 mM dithiothreitol DTT and 15 mM EDTA at pH 5.5. Subsequently, 2.5 µM of the activated cathepsin B was added to 25 µM of MMAE.VC.SA.617 and incubated at 37 °C. Aliquots were taken at different time points. The enzymatic activity of cathepsin B was blocked by adding 1 µL of E-64 (1 mM) in each vial. Samples were analyzed using an Agilent Technologies 1220 Infinity LC system coupled to an Agilent Technologies 6130B Single Quadrupole LC/MS system.

### 4.7. Animal Studies

Animals used for the biodistribution studies were obtained from the breeding facilities of the Institute of Biosciences and Applications, NCSR “Demokritos”. This experimental animal facility is registered according to the Greek Presidential Decree 56/2013 (Reg. Number: EL 25 BIO 022), in accordance with the European Directive 2010/63, which is harmonized with national legislation, on the protection of animals used for scientific purposes. All applicable national guidelines for the care and use of animals were followed. The study protocol was approved by the Department of Agriculture and Veterinary Service of the Prefecture of Athens.

#### 4.7.1. Toxicology Study in Healthy NOD/SCID Mice

Prior to the therapeutic efficacy study in LNCaP tumor-bearing mice, a toxicology study was carried out on healthy NOD/SCID mice to determine the tolerable dose of the single drug MMAE. Nine NOD/SCID mice were divided into 3 groups and received a single intravenous (i.v.) dose of MMAE. Group A received 0.1 mg/kg body weight. Group B received 0.5 mg/kg body weight, while Group C received 1 mg/kg body weight). Mice injected with 1 mg/kg MMAE had to be euthanized 5 days post-injection due to body-weight loss.

#### 4.7.2. Therapeutic Efficacy Study in LNCaP Tumor-Bearing NOD/SCID Mice

A therapeutic efficacy study of the MMAE.VC.SA.617 vs. MMAE was performed on six groups of LNCaP tumor-bearing NOD/SCID mice, two of which acted as reference groups, while an additional group acted as the control group. LNCaP cells were cultured in RPMI-1640 medium of pH 7.4, supplemented with 10% FBS, 100 U/mL of penicillin, 100 μg/mL of streptomycin, 2 mM glutamine, 10 mM HEPES and 1 mM sodium pyruvate. Cell cultures were maintained in 75 cm^2^ flasks, grown at 37 °C in 5% CO_2_ in a humidified atmosphere and the medium was changed approximately every 72 h (cell doubling time is about 40 h). Cells in the exponential phase of their growth were harvested by a 10 min treatment with a 0.05% trypsin–0.02% EDTA solution and neutralized with medium containing serum immediately. Cultures at passages 8–10 were used for the experiments. For the LNCaP xenograft development, cells were suspended in 100 μL in RPMI-1640 medium (supplemented as described above) and 100 μL Matrigel (medium: Matrigel ratio 1:1) (1 × 10^6^ cells/200 μL) and maintained on ice until the inoculation. All equipment (syringes and needles) was chilled on ice prior to use in tumor cell inoculation. The mice were subcutaneously inoculated under the left shoulder with the LNCaP cells. The animals were ready for experimentation approximately 14 days after cell inoculation, when the tumor reached a volume of about 300 mm^3^. Mice were randomly divided into six groups, as follows:

1. Group A: MMAE 0.1 mg/kg body weight (reference group A)
2. Group B: MMAE 0.5 mg/kg body weight (reference group B)
3. Group C: MMAE.VC.SA.617, 0.1 mg/kg body weight
4. Group D: MMAE.VC.SA.617, 0.5 mg/kg body weight
5. Group E: MMAE.VC.SA.617, 1.0 mg/kg body weight
6. Group F: Saline (control group)

All groups of mice were intravenously injected twice a week, for the first 5 doses of MMAE, MMAE.VC.SA.617 or saline, and then an additional 3 doses once a week, resulting in a total of 8 doses over a period of 7 weeks (100 μL injected volume per intravenous injection). Body weight and tumor volume were assessed on each day of intravenous injection, and every 3–4 days after the end of treatment administration, up to 63 days after the initiation of the therapeutic efficacy study (only the mice of Group E were monitored until day 63). Tumor volume was measured using calipers, and was calculated using the formula (length × width^2^)/2 [43,44]. The tumor growth index (TGI) for both animal groups was calculated by dividing the tumor volume measured each day by the initial tumor volume on day 0, before initiation of treatment. TGI was plotted vs. treatment time post-injection.

#### 4.7.3. Histology and Immunohistochemistry Staining

The tumors were fixed in 10% neutral buffered formalin (NBF, Sigma) and then routinely processed and paraffin embedded. Tumor sections were dewaxed and rehydrated and were then stained with hematoxylin and eosin (H&E). For immunohistochemistry, sections were antigen-retrieved with heat-induced or enzymatic method. Peroxidase activity was blocked using 1.5% hydrogen peroxide. Sections were blocked with different blocking protocols, depending on the antibody. Staining was performed using the following anti–mouse antibodies: anti-Ki67 (Cell Signaling, Danvers, MA, 01923 USA, REF 9449) (1:1000 dilution) and anti-Caspase 3 (Cell Signaling, 9661) (1:800 dilution). A polymer-based detection kit, which consisted of horseradish peroxidase–conjugated polymers was used for the detection. To determine proliferation indices, Ki67-positive and Ki67-negative cells were counted using ImageJ software (https://imagej.net/software/fiji/downloads) in 8–10 representative fields of all the tumors (on average, ~3000 nuclei were counted per specimen). A similar approach was followed in order to evaluate the % percentage of apoptotic cells.

## 5. Conclusions

In summary, we developed a novel SMDC for the treatment of PSMA-positive PC, which showed promising properties for a translational approach. The synthesis of MMAE.VC.SA.617, using squaric acid diester, was straightforward and carried out in only two steps. Concerning the pharmacokinetic profile of MMAE.VC.SA.617, we demonstrated the high PSMA-selectivity of this SMDC in vitro together with a cathepsin B specific cleavage. Both characteristics are crucial in the design of targeted drug conjugates. MMAE.VC.SA.617 showed in vitro, as well as in vivo, a cytotoxicity in the nanomolar range, based on the release of MMAE and its subsequent interaction with microtubules. Interestingly, although the in vitro cytotoxic effect of the developed conjugate was lower than the single drug MMAE, SMDC MMAE.VC.SA.617 clearly showed its superiority in vivo.

Even though the results reported here are highly promising, further optimization of the chemical structure of the MMAE–SMDC is required to improve the PSMA binding affinity and the cytotoxicity of the compound. A possible approach would be the use of a targeting vector with a higher PSMA binding affinity than KuE-617. On the other hand, the use of a different linker/spacer could either enhance the lipophilicity of the molecule and, thus, the passive diffusion through the cell membrane, or avoid possible interaction of MMAE with the targeting unit, which could lead to a change in the conformation of the SMDC and, therefore, to a disadvantageous orientation within the PSMA binding pocket.

## Figures and Tables

**Table 1:** PSMA drug conjugates that are either preclinically or clinically investigated.

PSMA Drug Conjugates		Targeting Unit	Payload	Status
**PSMA-ADCs**	MLN2704	J591	maytansinoid 1	discontinued at phase 2
	PSMA-MMAE	IgG1	MMAE	discontinued at phase 2
	MEDI3726	IgG1	pyrrolobenzodiazepine	discontinued at phase 1
**PSMA-SMDCs**	DUPA-SMDC	DUPA	indenoisoquinoline topoisomerase I inhibitor	preclinical studies
	DUPA-PTX	DUPA	paclitaxel	preclinical studies
	EC1169	KuE	tubulysin B	phase 1 NCT02202447
	PSMA-1-VcMMAE	PSMA-1	MMAE	preclinical studies
	SBPD-1	KuE	MMAE	preclinical studies

**Table 2:** Binding potency of the PSMA-targeted compounds. Values are expressed as mean ± SD.

Compound	IC_50_ [nM]
PSMA-11	17.4 ± 1.6
PSMA-617	15.1 ± 3.8
KuE-617	21.5 ± 1.9
MMAE.VC.SA.617	188.6 ± 24.7

**Table 3:** IC_50_ values of the compounds tested in the Celltiter-Blue^®^ cytotoxic assay. Values are mean ± SD.

Compound	IC_50_ [nM]
MMAE	0.23 ± 0.06
MMAE.VC.SA.617	33.0 ± 4.9
MMAE.VC.SA.617 + PMPA	92.8 ± 8.3
MMAE.VC.SA.617 + E-64	84.4 ± 0.1
